# Secular Trends and Prognostic Value of the Body Shape Index in U.S. Adults

**DOI:** 10.1002/osp4.70148

**Published:** 2026-04-24

**Authors:** Shuang Li, Yuchen Zhong, Yan Liu, Meixia Xiao, Shengming Shi

**Affiliations:** ^1^ Department of General Practice The First Affiliated Hospital of Huzhou Normal University Huzhou First People's Hospital Huzhou Zhejiang China; ^2^ Department of Medical Oncology Cancer Center Zhejiang Provincial People's Hospital Affiliated People's Hospital Hangzhou Medical College Hangzhou Zhejiang China

**Keywords:** Body Shape Index, cross‐sectional study, mortality, NHANES, obesity

## Abstract

**Objective:**

A Body Shape Index (BSI), previously developed and evaluated as an improvement over traditional adiposity measures for predicting health risks, has not been comprehensively characterized with respect to its temporal trends, population disparities, and prognostic value in the United States.

**Methods:**

This analysis examined data from 47,762 adults across 10 NHANES cycles (1999–2018). BSI trends were assessed across demographic and health‐related subgroups. All‐cause mortality risk was evaluated using Cox regression, with adjustment for multiple confounders. Interaction and restricted cubic spline analyses explored effect modification and nonlinearity.

**Results:**

Mean BSI increased significantly over the two decades. Disparities persisted or widened over time. Elevated BSI was independently associated with higher mortality risk, particularly above the median, with a nonlinear escalation in risk at higher BSI levels. The impact of high BSI on all‐cause mortality was stronger in males and varied by race/ethnicity, while lower BSI showed no protective effect.

**Conclusions:**

BSI demonstrates a rising trend and persistent disparities among U.S. adults, with high BSI independently predicting adverse all‐cause mortality in a nonlinear manner. These findings from the analyzed data supported the utility of BSI in risk stratification and highlighted the need for targeted interventions. Future research should explore BSI's role in public health strategies.

## 1 Introduction

Obesity and excess adiposity are recognized as multifaceted risk factors contributing to a spectrum of adverse outcomes, encompassing not only physical health issues such as cardiovascular disease, type 2 diabetes, and elevated mortality but also psychological dimensions including body image dissatisfaction, weight stigma, low self‐esteem, and mental health disorders such as depression and anxiety [1, 2, 3, 4, 5, 6, 7, 8, 9, 10, 11]. Recent literature highlights the bidirectional nature of these relationships: obesity can exacerbate negative body image and emotional distress through societal stigma and internalized bias, while poor body image and related behaviors, such as emotional eating or disordered eating patterns influenced by social media, may perpetuate weight gain and hinder effective management [12, 13]. For instance, body dissatisfaction has been shown to mediate the link between increasing BMI trajectories and diminished quality of life, with stronger effects in women due to sociocultural pressures emphasizing thinness [14]. Weight stigma, experienced by 40%–60% of individuals with overweight or obesity, emerges as a more significant driver of psychosocial distress than body size alone, contributing to higher rates of anxiety, depression, and loneliness, and potentially overshadowing direct physiological impacts [15, 16].

Traditionally, anthropometric indices such as body mass index (BMI) and waist circumference have been widely used to quantify adiposity and assess associated health risks at the population level [17, 18]. However, BMI's limitations are well‐documented in recent reviews: it fails to differentiate fat distribution, muscle mass, or ethnicity‐specific variations, potentially misclassifying individuals—such as those with normal BMI but high central fat—as low‐risk, while overlooking the metabolic dangers of visceral adiposity [18, 19]. Similarly, unadjusted waist circumference may not fully account for overall body proportions, leading to inaccuracies in risk assessment [20, 21].

To address these gaps, the Body Shape Index (BSI)—a metric adjusting waist circumference for height and BMI—has gained traction as a refined indicator of body shape, emphasizing central adiposity linked to insulin resistance, inflammation, cardiometabolic diseases, and even depressive symptoms [22, 23]. By scaling waist circumference to overall body size, BSI better isolates central (visceral) adiposity—the depot most strongly linked to insulin resistance, systemic inflammation, and cardiometabolic disease—thereby offering greater robustness than BMI or unadjusted waist circumference. Emerging evidence also connects higher BSI to greater depressive symptoms, suggesting a bridge between physical body shape and psychological body image concerns. Despite increasing interest in BSI, population‐based data on its secular trends, sociodemographic disparities, and prognostic implications remain limited, particularly in the United States. Understanding the temporal evolution of BSI and its distribution across diverse subgroups is essential for informing clinical risk stratification and designing effective public health interventions. Moreover, clarifying the association between BSI and all‐cause mortality, including possible effect modification by demographic and clinical characteristics, is critical for establishing its validity as a predictive biomarker.

Longitudinal data on national trends in BSI are essential to understand evolving patterns of body composition and associated disease burden. Previous studies utilizing the National Health and Nutrition Examination Survey (NHANES) have documented trends in BMI and waist circumference [24, 25, 26], but comprehensive analyses specifically focusing on BSI trends across demographic, socioeconomic, and health subgroups over extended periods are less established. Leveraging data from 10 consecutive cycles of NHANES spanning 1999 to 2018, this analysis aims to address these critical gaps with three primary objectives: (1) To characterize the temporal trends in mean BSI among US adults over this two‐decade period; (2) To examine disparities in BSI trends across key sociodemographic (age, sex, race/ethnicity, education, income) and health‐related (smoking, alcohol use, hypertension, diabetes) characteristics, assessing whether these disparities have persisted or widened; and (3) To rigorously evaluate the independent association between BSI levels and all‐cause mortality, we investigated the shape of the dose‐response relationship and potential effect modification by demographic and clinical factors. Understanding these dynamics is crucial for informing targeted public health interventions and refining risk stratification strategies based on body shape phenotypes.

## 2 Methods

### 2.1 Study Population and Data Source

This analysis utilized data from the NHANES, a series of cross‐sectional, nationally representative surveys conducted by the National Center for Health Statistics (NCHS) in the United States [27, 28, 29]. Data were pooled from 10 consecutive NHANES cycles spanning 1999–2018. A total of 101,316 participants were initially included from the pooled NHANES dataset. First, 1722 participants with pregnancy were excluded, yielding in 99,594 eligible participants. Next, 46,054 individuals under 20 years of age were excluded, leaving 53,540 adults aged 20 years and older. Subsequently, 5568 participants with missing waist circumference, height, and weight data were removed, yielding a final analytic sample of 47,762 participants. Missing data on these exposure components were handled via complete‐case exclusion; primary analyses assumed missing at random conditional on observed covariates, and no imputation was performed.

Mortality status and follow‐up time were ascertained by linkage to the National Death Index (NDI) [30], allowing for a robust assessment of all‐cause mortality. NHANES employs a complex multistage probability sampling design to ensure the representativeness of the U.S. civilian non‐institutionalized population. All participants provided informed consent, and the survey protocols were approved by the NCHS Institutional Review Board.

### 2.2 Anthropometric Measurements and BSI Calculation

Anthropometric data, including BMI, height, and waist circumference, were measured by trained examiners using standardized protocols. The BSI was calculated for each participant using the following modified formula:

$$\text{BSI}=\frac{\text{Waist}\;\text{circumference}\;\left(\text{cm}\right)}{{\text{BMI}\;}^{2/3}\times {\text{Height}}^{1/2}\;\left(m\right)}.$$

Notably, unlike the original BSI formula that uses waist circumference in meters, the present specification uses waist circumference in centimeters to improve interpretability and align with clinical practice. This adjustment does not affect the underlying mathematical relationship or the validity of the index, as all relevant anthropometric measures were uniformly converted and analyzed in centimeters. BSI quintiles were determined based on the distribution within each survey cycle.

### 2.3 Covariates and Subgroup Definitions

Sociodemographic variables included age, sex, race/ethnicity, education level, and poverty income ratio (PIR). Health‐related characteristics comprised smoking status (never, ever, current), alcohol use (categorized by frequency per week), hypertension, diabetes, and companion status (marital/living arrangement). Definitions for diabetes and hypertension were based on self‐report, medication use, or measured values according to NHANES protocols. Subgroups for stratified analyses were defined according to these characteristics.

### 2.4 Statistical Analysis

Descriptive statistics were used to summarize the distributions of BMI, height, waist circumference, and BSI. Secular trends in mean BSI across NHANES cycles were analyzed using weighted linear regression, accounting for complex survey design and sampling weights. Differences in mean BSI across demographic and health‐related subgroups were assessed using survey‐weighted estimation procedures. Temporal trends within subgroups were evaluated using interaction terms and stratified analyses.

To evaluate the association between BSI and all‐cause mortality, Cox proportional hazards regression models were employed, with follow‐up time calculated from the date of NHANES survey participation to the date of death or censoring. Hazard ratios (HRs) and 95% confidence intervals (CIs) were estimated for BSI quintiles, using the third quintile (Q3—the middle quintile, representing mid‐range BSI values) as the reference category. Four hierarchical adjustment models were constructed to assess the robustness of the observed associations: Model 1 (Unadjusted) included no covariates; Model 2 (Demographic‐adjusted) was adjusted for age and sex; Model 3 (Socioeconomic‐adjusted) further included race/ethnicity, education level, and poverty‐to‐income ratio; and Model 4 (Full model) additionally adjusted for marital status, smoking status, alcohol consumption, hypertension, diabetes, and the presence of obesity.

Interaction analyses were conducted to evaluate whether the association between BSI and all‐cause mortality differed across key subgroups, including age, sex, race/ethnicity, socioeconomic status, and clinical characteristics. Multiplicative interaction terms between BSI quintiles and subgroup variables were included in the fully adjusted model, and Wald tests were used to assess statistical significance.

To explore potential nonlinear associations between BSI and all‐cause mortality, restricted cubic spline (RCS) models were fitted in fully adjusted Cox models. The overall and nonlinear significance of the dose‐response relationship was evaluated using likelihood ratio tests. All analyses incorporated appropriate NHANES sample weights, strata, and primary sampling units to ensure nationally representative estimates. Statistical significance was defined as a two‐sided *p* value < 0.05. Analyses were conducted using R (version 4.2.0) [31].

## 3 Results

### 3.1 Temporal Trends in Body Shape Index Across NHANES Cycles

The distributions of BMI, height, waist circumference, and BSI among the analysis participants are shown in Supporting Information S1: Figure S1. BMI values ranged from 12.04 to 84.87, height from 123.3 to 204.5 cm, and waist circumference from 55.5 to 178.2 cm. BSI values, in contrast, exhibited a relatively narrow range (5.76–11.66) and demonstrated a distribution that closely approximated normality, with most participants clustering tightly around the mean. This contrasts with the broader, more right‐skewed distribution observed for BMI. The unimodal and symmetric distributions of all four anthropometric measures further support the representativeness and quality of the NHANES sample for analyses related to body composition and BSI.

**FIGURE 1:**
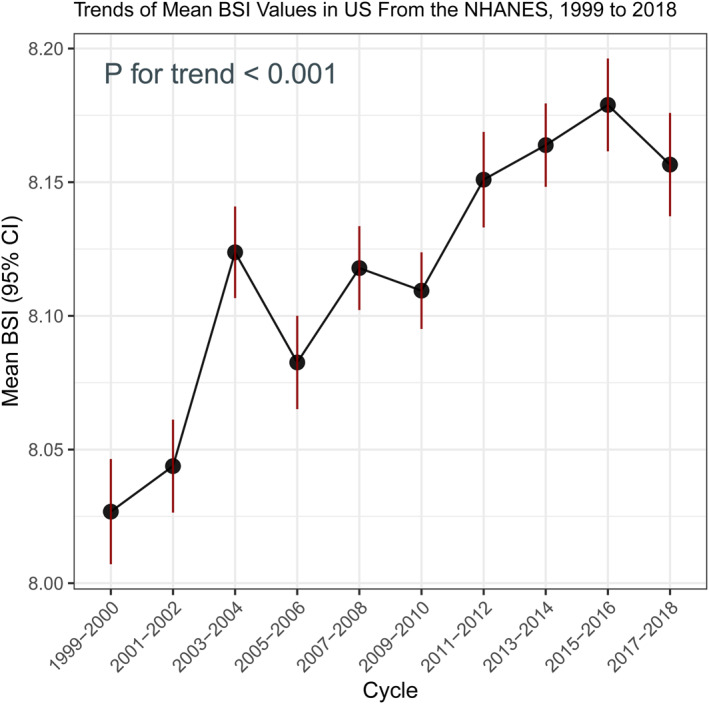
Trends in mean BSI values among US adults from 1999 to 2018 using NHANES data. The solid line represents mean BSI for each survey cycle, with error bars indicating 95% confidence intervals. The trend test was statistically significant (*p* for trend < 0.001).

The temporal analysis of the BSI among U.S. adults, based on consecutive NHANES survey cycles from 1999 to 2018, revealed notable fluctuations and an overall upward trend during the analysis period (Table 1 and Figure 1). The mean BSI in 1999–2000 was 8.10 (SE = 0.008), with the value remaining relatively stable in 2001–2002 (mean = 8.11, SE = 0.008; *p* = 0.48 vs. 1999–2000). A significant increase was observed in 2003–2004 (mean = 8.19, *p* < 0.001), followed by a slight decline in 2005–2006 (mean = 8.12, *p* = 0.049) and another significant rise in 2007–2008 (mean = 8.17, *p* < 0.001).

**TABLE 1:** Weighted mean Body Shape Index and 95% confidence intervals among U.S. adults across consecutive NHANES cycles, 1999–2018.

Year	Mean	SE	Lower CI	Upper CI	Difference	*p* value
1999–2000	8.098	0.008365	8.081	8.114	0.000	NA
2001–2002	8.106	0.007955	8.090	8.122	0.008	0.483
2003–2004	8.187	0.007949	8.172	8.203	0.089	< 0.0001
2005–2006	8.121	0.007927	8.105	8.136	0.023	0.049
2007–2008	8.174	0.006892	8.161	8.188	0.076	< 0.0001
2009–2010	8.156	0.006365	8.144	8.169	0.058	< 0.0001
2011–2012	8.146	0.006946	8.132	8.160	0.048	< 0.0001
2013–2014	8.179	0.006632	8.166	8.192	0.081	< 0.0001
2015–2016	8.197	0.006876	8.184	8.211	0.100	< 0.0001
2017–2018	8.193	0.007203	8.179	8.208	0.096	< 0.0001

*Note:* Differences and *p*‐values are calculated relative to the 1999–2000 cycle.

Subsequent cycles demonstrated variations, and the mean BSI values generally exhibited a gradual increase over time. Notably, the mean BSI in 2015–2016 (8.20) and 2017–2018 (8.19) were both significantly higher compared to the baseline cycle (*p* < 0.001 for both comparisons). The confidence intervals for each survey cycle were narrow, indicating a high precision of the estimates across the 20‐year span.

These findings indicate a discernible upward trend in BSI among U.S. adults over the past two decades, suggesting a shift in body shape characteristics at the population level.

### 3.2 Subgroup Trends in BSI Across Sociodemographic and Health Characteristics

**FIGURE 2:**
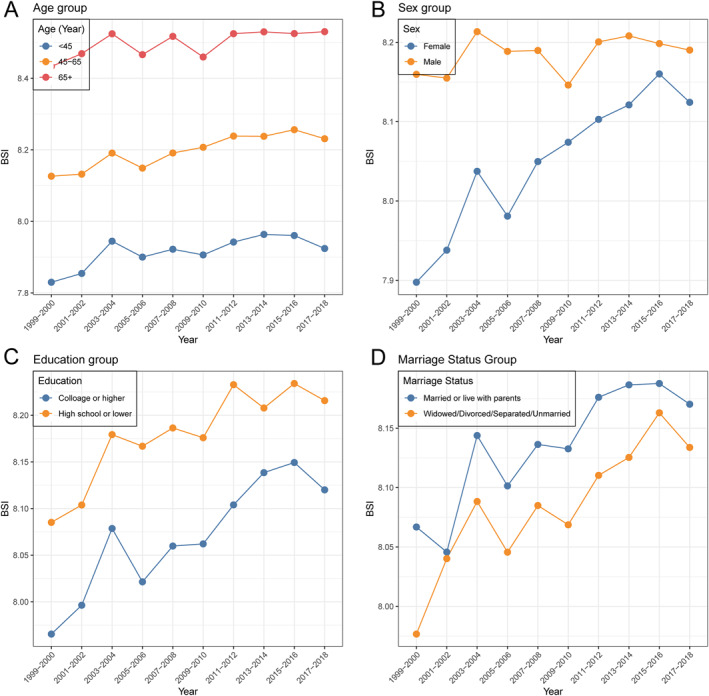
Trends in mean BSI among U.S. adults by (A) age group, (B) sex, (C) education, and (D) marital status, 1999–2018.

**FIGURE 3:**
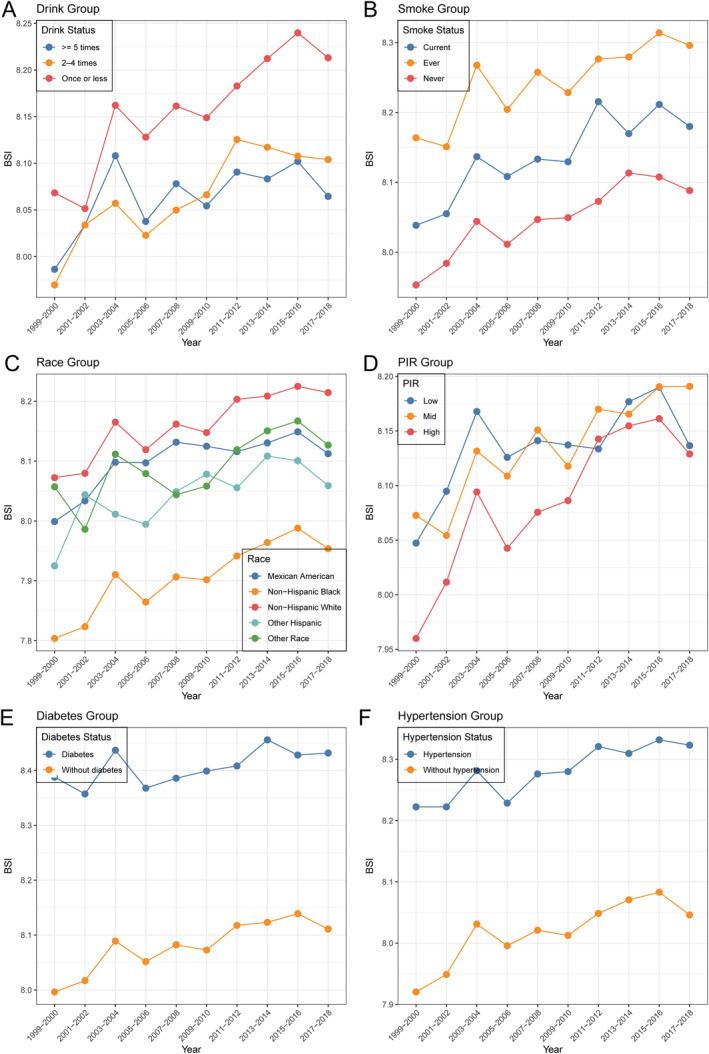
Trends in mean BSI among U.S. adults by (A) drinking status, (B) smoking status, (C) race/ethnicity, (D) poverty income ratio, (E) diabetes status, and (F) hypertension status, 1999–2018.

Subgroup analyses revealed persistent and widening disparities in BSI across demographic, socioeconomic, and health‐related characteristics from 1999 to 2018 (Table 2; Figures 2, 3). Among age groups, the highest mean BSI was consistently observed in adults aged 65 years and older, increasing from 8.43 (95% CI: 8.40–8.47; *n* = 1116) in 1999–2000 to 8.53 (95% CI: 8.49–8.57; *n* = 1255) in 2017–2018, while the < 45 age group maintained the lowest BSI throughout the analysis period. Males had higher mean BSI values than females in all cycles, with both sexes demonstrating gradual upward trends.

**TABLE 2:** Weighted mean Body Shape Index and 95% confidence intervals by demographic, socioeconomic, lifestyle, and health status subgroups across NHANES cycles, 1999–2018.

Var	Subgroup	1999–2000	2001–2002	2003–2004	2005–2006	2007–2008	2009–2010	2011–2012	2013–2014	2015–2016	2017–2018
Diabetes	Diabetes	8.39 (8.33, 8.44); *n* = 489	8.36 (8.3, 8.41); *n* = 497	8.44 (8.39, 8.48); *n* = 581	8.37 (8.32, 8.42); *n* = 558	8.39 (8.35, 8.43); *n* = 894	8.4 (8.36, 8.44); *n* = 865	8.41 (8.36, 8.46); *n* = 788	8.46 (8.42, 8.49); *n* = 816	8.43 (8.39, 8.47); *n* = 912	8.43 (8.39, 8.48); *n* = 980
	Without diabetes	8 (7.98, 8.02); *n* = 3531	8.02 (8, 8.04); *n* = 3850	8.09 (8.07, 8.11); *n* = 3666	8.05 (8.03, 8.07); *n* = 3620	8.08 (8.07, 8.1); *n* = 4417	8.07 (8.06, 8.09); *n* = 4764	8.12 (8.1, 8.14); *n* = 4118	8.12 (8.11, 8.14); *n* = 4380	8.14 (8.12, 8.16); *n* = 4132	8.11 (8.09, 8.13); *n* = 3904
Hypertension	Hypertension	8.22 (8.19, 8.25); *n* = 1737	8.22 (8.19, 8.25); *n* = 1761	8.28 (8.25, 8.31); *n* = 1881	8.23 (8.2, 8.26); *n* = 1713	8.28 (8.25, 8.3); *n* = 2362	8.28 (8.26, 8.3); *n* = 2358	8.32 (8.29, 8.35); *n* = 2060	8.31 (8.28, 8.33); *n* = 2198	8.33 (8.31, 8.36); *n* = 2179	8.32 (8.29, 8.35); *n* = 2277
	Without hypertension	7.92 (7.9, 7.94); *n* = 2283	7.95 (7.93, 7.97); *n* = 2586	8.03 (8.01, 8.05); *n* = 2366	8 (7.97, 8.02); *n* = 2465	8.02 (8, 8.04); *n* = 2949	8.01 (7.99, 8.03); *n* = 3271	8.05 (8.03, 8.07); *n* = 2846	8.07 (8.05, 8.09); *n* = 2998	8.08 (8.06, 8.11); *n* = 2865	8.05 (8.02, 8.07); *n* = 2607
Drink weekly	More than 5 times	7.99 (7.94, 8.03); *n* = 605	8.03 (8, 8.07); *n* = 641	8.11 (8.07, 8.15); *n* = 594	8.04 (7.99, 8.08); *n* = 612	8.08 (8.04, 8.11); *n* = 807	8.05 (8.02, 8.09); *n* = 929	8.09 (8.05, 8.13); *n* = 720	8.08 (8.04, 8.12); *n* = 697	8.1 (8.06, 8.14); *n* = 697	8.06 (8.02, 8.11); *n* = 592
	2–5 times	7.97 (7.93, 8.01); *n* = 980	8.03 (8, 8.07); *n* = 1102	8.06 (8.02, 8.09); *n* = 1043	8.02 (7.99, 8.05); *n* = 1103	8.05 (8.02, 8.08); *n* = 1289	8.07 (8.04, 8.09); *n* = 1457	8.13 (8.09, 8.16); *n* = 1251	8.12 (8.09, 8.15); *n* = 1429	8.11 (8.07, 8.14); *n* = 1346	8.1 (8.07, 8.14); *n* = 1392
	Once or less	8.07 (8.04, 8.09); *n* = 2435	8.05 (8.03, 8.08); *n* = 2604	8.16 (8.14, 8.19); *n* = 2610	8.13 (8.1, 8.15); *n* = 2463	8.16 (8.14, 8.18); *n* = 3215	8.15 (8.13, 8.17); *n* = 3243	8.18 (8.16, 8.21); *n* = 2935	8.21 (8.19, 8.23); *n* = 3070	8.24 (8.22, 8.26); *n* = 3001	8.21 (8.19, 8.24); *n* = 2900
Smoke status	Current	8.04 (8, 8.08); *n* = 853	8.06 (8.02, 8.09); *n* = 1017	8.14 (8.1, 8.17); *n* = 996	8.11 (8.07, 8.14); *n* = 955	8.13 (8.1, 8.16); *n* = 1213	8.13 (8.1, 8.16); *n* = 1227	8.22 (8.18, 8.25); *n* = 1002	8.17 (8.14, 8.2); *n* = 1070	8.21 (8.17, 8.25); *n* = 966	8.18 (8.14, 8.22); *n* = 900
	Ever	8.16 (8.13, 8.2); *n* = 1081	8.15 (8.12, 8.19); *n* = 1144	8.27 (8.24, 8.3); *n* = 1154	8.2 (8.17, 8.24); *n* = 1065	8.26 (8.22, 8.29); *n* = 1320	8.23 (8.2, 8.26); *n* = 1375	8.28 (8.24, 8.31); *n* = 1105	8.28 (8.25, 8.31); *n* = 1201	8.31 (8.28, 8.35); *n* = 1155	8.3 (8.26, 8.33); *n* = 1168
	Never	7.95 (7.93, 7.98); *n* = 2086	7.98 (7.96, 8.01); *n* = 2186	8.04 (8.02, 8.07); *n* = 2097	8.01 (7.99, 8.04); *n* = 2158	8.05 (8.03, 8.07); *n* = 2778	8.05 (8.03, 8.07); *n* = 3027	8.07 (8.05, 8.1); *n* = 2799	8.11 (8.09, 8.13); *n* = 2925	8.11 (8.09, 8.13); *n* = 2923	8.09 (8.06, 8.11); *n* = 2816
PIR	Low	8.05 (8, 8.09); *n* = 1066	8.09 (8.06, 8.13); *n* = 1054	8.17 (8.13, 8.2); *n* = 1148	8.13 (8.09, 8.16); *n* = 1020	8.14 (8.11, 8.17); *n* = 1484	8.14 (8.11, 8.16); *n* = 1705	8.13 (8.1, 8.16); *n* = 1621	8.18 (8.15, 8.2); *n* = 1632	8.19 (8.16, 8.22); *n* = 1439	8.14 (8.1, 8.17); *n* = 1205
	Mid	8.07 (8.04, 8.1); *n* = 1881	8.05 (8.03, 8.08); *n* = 1860	8.13 (8.1, 8.16); *n* = 1846	8.11 (8.08, 8.14); *n* = 1760	8.15 (8.13, 8.18); *n* = 2347	8.12 (8.1, 8.14); *n* = 2445	8.17 (8.14, 8.2); *n* = 1916	8.17 (8.14, 8.19); *n* = 2047	8.19 (8.16, 8.22); *n* = 2313	8.19 (8.16, 8.22); *n* = 2375
	High	7.96 (7.93, 7.99); *n* = 1073	8.01 (7.98, 8.04); *n* = 1433	8.09 (8.07, 8.12); *n* = 1253	8.04 (8.01, 8.07); *n* = 1398	8.08 (8.05, 8.1); *n* = 1480	8.09 (8.06, 8.11); *n* = 1479	8.14 (8.11, 8.17); *n* = 1369	8.15 (8.13, 8.18); *n* = 1517	8.16 (8.13, 8.19); *n* = 1292	8.13 (8.1, 8.16); *n* = 1304
Education level	Collage or higher	7.97 (7.94, 7.99); *n* = 1526	8 (7.97, 8.02); *n* = 2029	8.08 (8.06, 8.1); *n* = 1935	8.02 (8, 8.05); *n* = 2042	8.06 (8.04, 8.08); *n* = 2359	8.06 (8.04, 8.08); *n* = 2740	8.1 (8.08, 8.13); *n* = 2743	8.14 (8.12, 8.16); *n* = 2937	8.15 (8.13, 8.17); *n* = 2774	8.12 (8.09, 8.15); *n* = 2762
	High school or lower	8.09 (8.06, 8.11); *n* = 2494	8.1 (8.08, 8.13); *n* = 2318	8.18 (8.15, 8.2); *n* = 2312	8.17 (8.14, 8.19); *n* = 2136	8.19 (8.17, 8.21); *n* = 2952	8.18 (8.16, 8.2); *n* = 2889	8.23 (8.21, 8.26); *n* = 2163	8.21 (8.18, 8.23); *n* = 2259	8.23 (8.21, 8.26); *n* = 2270	8.22 (8.19, 8.25); *n* = 2122
Race and ethnicity	Mexican American	8 (7.97, 8.03); *n* = 1087	8.03 (8.01, 8.06); *n* = 913	8.1 (8.07, 8.13); *n* = 847	8.1 (8.07, 8.13); *n* = 817	8.13 (8.1, 8.16); *n* = 911	8.12 (8.1, 8.15); *n* = 1043	8.12 (8.08, 8.15); *n* = 486	8.13 (8.1, 8.16); *n* = 700	8.15 (8.12, 8.18); *n* = 881	8.11 (8.07, 8.15); *n* = 646
	Non‐Hispanic Black	7.8 (7.77, 7.84); *n* = 763	7.82 (7.79, 7.86); *n* = 853	7.91 (7.88, 7.94); *n* = 836	7.86 (7.83, 7.9); *n* = 966	7.91 (7.88, 7.94); *n* = 1099	7.9 (7.87, 7.93); *n* = 976	7.94 (7.91, 7.97); *n* = 1282	7.96 (7.94, 7.99); *n* = 1050	7.99 (7.96, 8.02); *n* = 1050	7.95 (7.92, 7.99); *n* = 1148
	Non‐Hispanic White	8.07 (8.05, 8.1); *n* = 1793	8.08 (8.06, 8.1); *n* = 2272	8.17 (8.14, 8.19); *n* = 2253	8.12 (8.1, 8.14); *n* = 2101	8.16 (8.14, 8.18); *n* = 2490	8.15 (8.13, 8.17); *n* = 2721	8.2 (8.18, 8.23); *n* = 1799	8.21 (8.19, 8.23); *n* = 2226	8.22 (8.2, 8.25); *n* = 1669	8.21 (8.19, 8.24); *n* = 1694
	Other Hispanic	7.93 (7.86, 7.99); *n* = 253	8.04 (7.97, 8.12); *n* = 177	8.01 (7.92, 8.1); *n* = 130	7.99 (7.91, 8.08); *n* = 130	8.05 (8.01, 8.09); *n* = 595	8.08 (8.04, 8.12); *n* = 581	8.06 (8.01, 8.1); *n* = 504	8.11 (8.07, 8.15); *n* = 471	8.1 (8.06, 8.14); *n* = 682	8.06 (8.01, 8.11); *n* = 449
	Other race	8.06 (7.95, 8.16); *n* = 124	7.99 (7.9, 8.07); *n* = 132	8.11 (8.04, 8.18); *n* = 181	8.08 (8.01, 8.15); *n* = 164	8.04 (7.97, 8.11); *n* = 216	8.06 (8, 8.11); *n* = 308	8.12 (8.07, 8.17); *n* = 835	8.15 (8.11, 8.19); *n* = 749	8.17 (8.13, 8.21); *n* = 762	8.13 (8.08, 8.17); *n* = 947
Marriage status	Married or live with parents	8.07 (8.04, 8.09); *n* = 2204	8.05 (8.02, 8.07); *n* = 2738	8.14 (8.12, 8.17); *n* = 2540	8.1 (8.08, 8.12); *n* = 2603	8.14 (8.12, 8.16); *n* = 3198	8.13 (8.11, 8.15); *n* = 3369	8.18 (8.15, 8.2); *n* = 2783	8.19 (8.17, 8.21); *n* = 3094	8.19 (8.17, 8.21); *n* = 3074	8.17 (8.15, 8.19); *n* = 2891
	Widowed/divorced/separated/unmarried	7.98 (7.95, 8.01); *n* = 1816	8.04 (8.01, 8.07); *n* = 1609	8.09 (8.06, 8.12); *n* = 1707	8.05 (8.01, 8.08); *n* = 1575	8.08 (8.06, 8.11); *n* = 2113	8.07 (8.04, 8.09); *n* = 2260	8.11 (8.08, 8.14); *n* = 2123	8.13 (8.1, 8.15); *n* = 2102	8.16 (8.13, 8.19); *n* = 1970	8.13 (8.1, 8.17); *n* = 1993
Sex	Female	7.9 (7.87, 7.93); *n* = 2015	7.94 (7.91, 7.96); *n* = 2163	8.04 (8.01, 8.06); *n* = 2094	7.98 (7.95, 8.01); *n* = 2034	8.05 (8.03, 8.07); *n* = 2680	8.07 (8.05, 8.1); *n* = 2868	8.1 (8.08, 8.13); *n* = 2437	8.12 (8.1, 8.14); *n* = 2665	8.16 (8.13, 8.19); *n* = 2578	8.12 (8.1, 8.15); *n* = 2483
	Male	8.16 (8.14, 8.18); *n* = 2005	8.15 (8.13, 8.18); *n* = 2184	8.21 (8.19, 8.24); *n* = 2153	8.19 (8.17, 8.21); *n* = 2144	8.19 (8.17, 8.21); *n* = 2631	8.15 (8.13, 8.17); *n* = 2761	8.2 (8.18, 8.23); *n* = 2469	8.21 (8.19, 8.23); *n* = 2531	8.2 (8.18, 8.22); *n* = 2466	8.19 (8.16, 8.22); *n* = 2401
Age group	< 45	7.83 (7.8, 7.86); *n* = 1658	7.85 (7.83, 7.88); *n* = 1911	7.94 (7.92, 7.97); *n* = 1757	7.9 (7.88, 7.92); *n* = 1852	7.92 (7.9, 7.94); *n* = 2138	7.91 (7.89, 7.92); *n* = 2395	7.94 (7.92, 7.96); *n* = 2162	7.96 (7.94, 7.98); *n* = 2261	7.96 (7.94, 7.98); *n* = 2133	7.92 (7.9, 7.95); *n* = 1829
	45–65	8.13 (8.09, 8.16); *n* = 1246	8.13 (8.1, 8.16); *n* = 1404	8.19 (8.16, 8.22); *n* = 1258	8.15 (8.12, 8.18); *n* = 1325	8.19 (8.17, 8.22); *n* = 1850	8.21 (8.18, 8.23); *n* = 1918	8.24 (8.21, 8.27); *n* = 1729	8.24 (8.21, 8.26); *n* = 1813	8.26 (8.23, 8.28); *n* = 1764	8.23 (8.2, 8.26); *n* = 1800
	65+	8.43 (8.4, 8.47); *n* = 1116	8.47 (8.43, 8.51); *n* = 1032	8.52 (8.49, 8.56); *n* = 1232	8.47 (8.43, 8.5); *n* = 1001	8.52 (8.49, 8.55); *n* = 1323	8.46 (8.43, 8.49); *n* = 1316	8.52 (8.48, 8.56); *n* = 1015	8.53 (8.5, 8.56); *n* = 1122	8.52 (8.49, 8.56); *n* = 1147	8.53 (8.49, 8.57); *n* = 1255

Regarding race and ethnicity, Non‐Hispanic White participants consistently exhibited the highest mean BSI, followed by Mexican American and “Other Race” groups, while Non‐Hispanic Black participants maintained the lowest values. These patterns, and the observed race/ethnicity interactions, may reflect differences in central versus subcutaneous fat distribution, cardiometabolic susceptibility at comparable body size, and contextual factors—including socioeconomic conditions, diet and physical activity, healthcare access, chronic stress, and environmental exposures—that jointly influence both BSI and mortality risk. Education and income gradients were evident: individuals with high school education or lower, and those in the lowest PIR group had higher BSI than their more advantaged counterparts, and these differences persisted over time.

Health behavior and status were also associated with BSI trajectories. Current and ever smokers exhibited a higher BSI than never smokers, while participants who drank once or less per week had higher BSI than those who drank more frequently. The presence of hypertension or diabetes was strongly associated with elevated BSI; for instance, participants with diabetes had a mean BSI of 8.39 (95% CI: 8.33–8.44; *n* = 489) in 1999–2000, compared with 8.00 (95% CI: 7.98–8.02; *n* = 3531) among those without diabetes, and this gap remained apparent across cycles. A similar pattern was observed for hypertension. Collectively, these findings underscore persistence and, in some cases, widening disparities in BSI across sociodemographic and health characteristics over the past two decades.

### 3.3 Association Between BSI and All‐Cause Mortality

Table 3 demonstrates the association between BSI quintiles and all‐cause mortality across different adjustment models. In the unadjusted analysis, higher BSI was strongly related to increased risk: compared with the reference group (Q3, the middle BSI quintile), the lowest quintile (Q1) had a significantly reduced risk (HR = 0.44, 95% CI: 0.39–0.50), while the highest quintile (Q5) had a markedly elevated risk (HR = 4.09, 95% CI: 3.72–4.49; *p* < 0.001). After adjustment for covariates in Model 1, the associations were attenuated but remained significant for Q2 (HR = 0.87, 95% CI: 0.79–0.97) and Q5 (HR = 1.50, 95% CI: 1.38–1.64). Further adjustment in Model 2 yielded similar results, and in the fully adjusted model, the highest BSI group (Q5) still exhibited a significantly increased risk (HR = 1.36, 95% CI: 1.24–1.49), while the associations for the lower quintiles became non‐significant. These findings suggest that elevated BSI is independently associated with worse prognosis, even after accounting for potential confounders.

**TABLE 3:** Association between BSI quintiles and prognosis (hazard ratios and 95% confidence intervals) in unadjusted and multivariable‐adjusted models.

Group	Q1	Q2	Q3	Q4	Q5
Without adjustment	0.44 (0.39, 0.50)***	0.61 (0.55, 0.68)***	1 [Ref]	1.70 (1.52, 1.89)***	4.09 (3.72, 4.49)***
Adjust model 1	0.88 (0.77, 1.01)	0.87 (0.79, 0.97)*	1 [Ref]	1.11 (1.00, 1.23)	1.50 (1.38, 1.64)***
Adjust model 2	0.87 (0.77, 0.99)*	0.88 (0.79, 0.97)*	1 [Ref]	1.09 (0.98, 1.22)	1.44 (1.31, 1.58)***
Adjust model fully	0.91 (0.79, 1.04)	0.91 (0.82, 1.01)	1 [Ref]	1.08 (0.97, 1.21)	1.36 (1.24, 1.49)***

*Note:* Q3 serves as the reference group.

*p < 0.05.

***p < 0.001.

### 3.4 Subgroup Analyses of the Association Between BSI and All‐Cause Mortality

**FIGURE 4:**
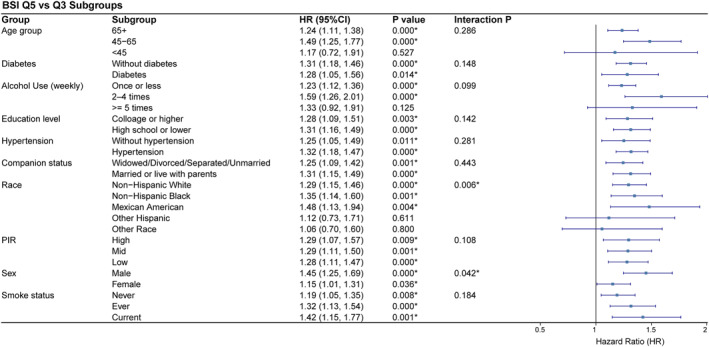
Subgroup analyses of the association between the highest quintile of BSI (Q5) versus the reference group (Q3) and adverse prognosis. Hazard ratios (HRs) and 95% confidence intervals are shown for each subgroup, along with *p*‐values and interaction *p*‐values for heterogeneity across subgroups.

Figure 4 presents the results of subgroup analyses comparing the highest BSI quintile (Q5) with the reference group (Q3, the middle BSI quintile; reference) across various demographic and clinical characteristics. Elevated BSI (Q5) was significantly associated with increased risk of adverse prognosis in nearly all subgroups, with HRs ranging from 1.15 (1.01–1.31) in females to 1.49 (1.25–1.77) in individuals aged 45–65 years. This association was consistently significant across strata of age, diabetes status, alcohol use, education level, hypertension, marital status, income, and smoking status.

Importantly, statistically significant interaction effects were found for both sex (*p* for interaction = 0.042) and race (*p* for interaction = 0.006), indicating that the prognostic impact of elevated BSI may be modified by these factors. Specifically, the association between high BSI and adverse prognosis was stronger in males (HR = 1.45, 95% CI: 1.25–1.69) than in females (HR = 1.15, 95% CI: 1.01–1.31). Similarly, among racial/ethnic subgroups, the risk estimates varied: Non‐Hispanic Black (HR = 1.35, 95% CI: 1.14–1.60) and Mexican American (HR = 1.48, 95% CI: 1.13–1.94) individuals showed a more pronounced association compared to Non‐Hispanic White (HR = 1.29, 95% CI: 1.15–1.46) and other groups. These findings suggest that sex and race both significantly influence the prognostic value of BSI, highlighting important heterogeneity in the association between BSI and health outcomes within the population.

Supporting Information S1: Figure S2 shows that there was no significant difference in all‐cause mortality between participants in the lowest BSI quintile (Q1) and the reference group (Q3, the middle BSI quintile; reference) across all examined subgroups. The hazard ratios for Q1 versus Q3 were close to 1, with 95% confidence intervals crossing 1 and no statistically significant associations observed in any subgroup. These findings indicate that lower BSI levels were not associated with a reduced risk of adverse outcomes.

### 3.5 Nonlinear Association Between BSI and All‐Cause Mortality

**FIGURE 5:**
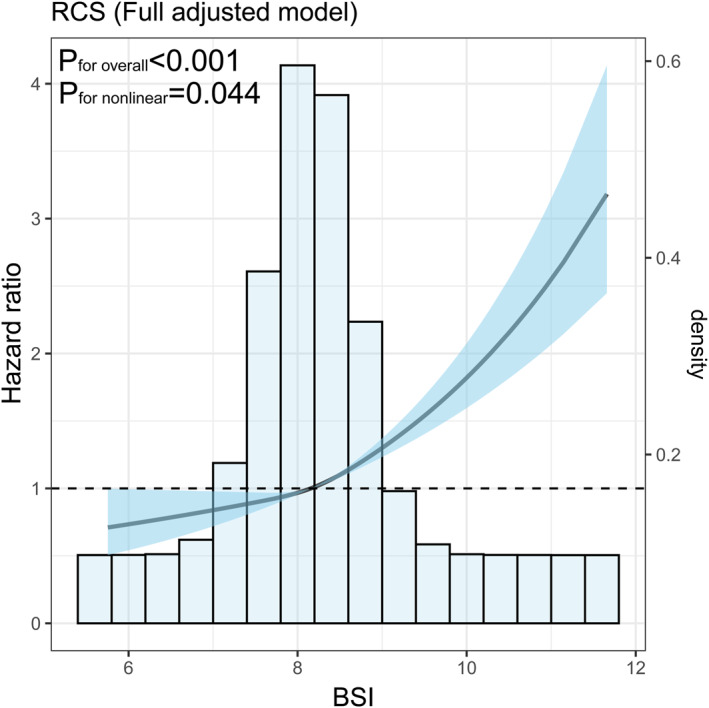
Restricted cubic spline analysis illustrating the nonlinear association between BSI and the hazard ratio for all‐cause mortality in the fully adjusted model. The solid line represents the estimated hazard ratio and the shaded area indicates the 95% confidence interval. The histogram displays the distribution of BSI in the study population. Both the overall and nonlinear effects were statistically significant.

The dose‐response relationship between BSI and all‐cause mortality was further investigated using a restricted cubic spline model (Figure 5). After full adjustment for potential confounders, the analysis revealed a significant overall association (*p* < 0.001) as well as a significant nonlinear pattern (*p* = 0.044). The hazard ratio remained close to 1 at lower BSI values, indicating no increased risk in this range. However, as BSI increased beyond the median, the hazard ratio rose rapidly, suggesting a strong and nonlinear escalation in risk associated with higher BSI levels.

These findings indicate that while low to moderate BSI levels are not associated with excess risk, higher BSI values confer a disproportionately greater risk of adverse outcomes. This nonlinear association underscores the importance of careful monitoring and intervention among individuals with elevated BSI.

## 4 Discussion

This comprehensive analysis of nationally representative NHANES data (1999–2018) reveals three principal findings: a significant upward temporal trend in BSI among U.S. adults over two decades; persistent sociodemographic, behavioral, and health‐related disparities in BSI distribution; and a robust, independent, nonlinear association between elevated BSI and adverse prognosis [22]. These observations merit careful interpretation within the methodological constraints of a cross‐sectional design, which inherently precludes causal inference.

The observed secular increase in mean BSI is consistent with broader trends in central adiposity and metabolic risk reported in recent U.S. population studies [32, 33, 34]. These findings align with a study using the same NHANES timeframe (1999–2018), which documented similar upward shifts in BSI linked to chronic kidney disease prevalence and mortality, suggesting sustained population‐level changes in body composition [35]. These findings further highlight that this upward trajectory in BSI is not evenly distributed, with older adults, males, individuals with lower socioeconomic status, and those with adverse health behaviors exhibiting consistently higher BSI values. These patterns suggest that population‐level interventions and targeted prevention strategies are needed to address the persistent and, in some cases, widening disparities in body shape and associated health risks.

The association between BSI and all‐cause mortality was most pronounced among those with the highest BSI quintile, and this relationship persisted across nearly all examined subgroups. The subgroup analyses further underscore persistent disparities, with older adults and males showing consistently higher BSI, mirroring 2025 findings where sex‐ and age‐specific variations in BSI were pronounced in diabetic cohorts, with males exhibiting stronger mortality links [36]. The nonlinear dose‐response pattern, characterized by a marked increase in risk above the population median, underscores the clinical relevance of BSI as a potential risk stratification tool, echoing findings of nonlinear thresholds in ABSI‐mortality links, such as *z*‐scores > 0.858 for all‐cause death [35]. Notably, the absence of a protective effect in the lowest BSI quintile suggests that only elevated, rather than reduced, BSI is of prognostic concern, a pattern observed in diabetic cohorts where low ABSI trajectories did not mitigate risks compared to moderate increases [37]. This may be because low BSI includes both healthy lean individuals and those with underlying illness or frailty, diluting any protective effect, as supported by U‐shaped mortality curves in related indices like body roundness index [38]. Unlike BMI, low central adiposity as measured by BSI may not increase mortality risk, reflecting its metabolic neutrality. Residual confounding or reverse causality, such as illness‐related weight loss, may also mask the potential benefits of low BSI. Overall, BSI appears most valuable for identifying risk at higher levels rather than at the lower end of the spectrum. The significant interactions observed for sex and race/ethnicity further suggest that the health consequences of altered body shape may be shaped by underlying biological, behavioral, or sociocultural factors, consistent with sex‐specific variations in ABSI‐osteoarthritis links and stronger mortality associations in males [39].

Despite the strengths of this analysis—including the large, nationally representative sample, standardized data collection, and rigorous adjustment for confounders—several limitations should be acknowledged. Several limitations necessitate cautious interpretation. First, as a cross‐sectional analysis of survey data, this analysis identifies associations but cannot infer causality or establish temporal sequences. While mortality follow‐up from the National Death Index enhances the prognostic relevance of these findings, residual confounding and reverse causation cannot be ruled out. Second, certain relevant variables, such as physical activity, dietary intake, and genetic factors, were not fully accounted for and may influence both BSI and health outcomes. Third, generalizability beyond the U.S. population is uncertain, particularly for regions with distinct body composition profiles.

This analysis further highlights the clinical and public health significance of BSI as an anthropometric predictor of health risk. Compared with traditional indices such as BMI and waist circumference, BSI demonstrates unique advantages in capturing central adiposity and predicting mortality risk. Notably, systematic reviews and meta‐analyses have shown that BSI outperforms BMI and waist circumference in predicting all‐cause mortality, though its performance for chronic disease prediction is less robust [23]. Prospective population‐based studies further indicate that BSI is more strongly associated with total, cardiovascular, and cancer mortality compared with other common anthropometric measures [40]. Despite these strengths, practical challenges remain, including BSI's highly clustered distribution and small variance, which complicate the establishment of clear clinical thresholds. Nonetheless, the demonstrated stability of BSI across survey cycles, its discriminatory power among subgroups, and its prognostic validity support its potential value as an adjunctive metric for assessing adiposity‐related health risks. Clinically, the observed nonlinear escalation in risk underscores the importance of targeting interventions—such as lifestyle modification—specifically to individuals with elevated BSI. Future research should explore the integration of BSI with traditional indices in screening, risk assessment, and chronic disease management, as well as its combination with lifestyle and genetic factors to enable precision prevention. Further longitudinal and interventional studies are warranted to validate BSI's predictive value for specific outcomes and to determine whether reducing BSI directly translates into lower health risks.

In conclusion, this analysis demonstrates a clear upward trend in BSI among U.S. adults over the past two decades, with persistent disparities across sociodemographic and health‐related characteristics. Elevated BSI is independently associated with increased risk of adverse prognosis, particularly at higher levels, and this relationship is modified by sex and race/ethnicity. While these findings highlight BSI as a promising anthropometric tool, they derive from observational data and thus reflect association and not causation. Future research should prioritize causal frameworks and intervention trials to clarify BSI's role in precision public health strategies.

## Author Contributions

**Shuang Li:** conceptualization, methodology, formal analysis, writing – original draft. **Yuchen Zhong:** conceptualization, data curation, software, writing – original draft. **Yan Liu:** data curation, investigation, validation. **Meixia Xiao:** methodology, visualization, writing – review and editing. **Shengming Shi:** supervision, project administration, funding acquisition, writing – review and editing.

## Funding

Huzhou Municipal Science and Technology Bureau General Project (Grant 2023GY40).

## Conflicts of Interest

The authors declare no conflicts of interest.

## Supporting information


Supporting Information S1
