# Supplementary material for: Secular Trends and Prognostic Value of the Body Shape Index in U.S. Adults
Source: Obes Sci Pract. 2026 Apr 24;12(2):e70148. doi: 10.1002/osp4.70148 (PMC13109581; doi:10.1002/osp4.70148)
Supplement: Supplementary file 1 — Supporting Information S1 [file OSP4-12-e70148-s001.docx]

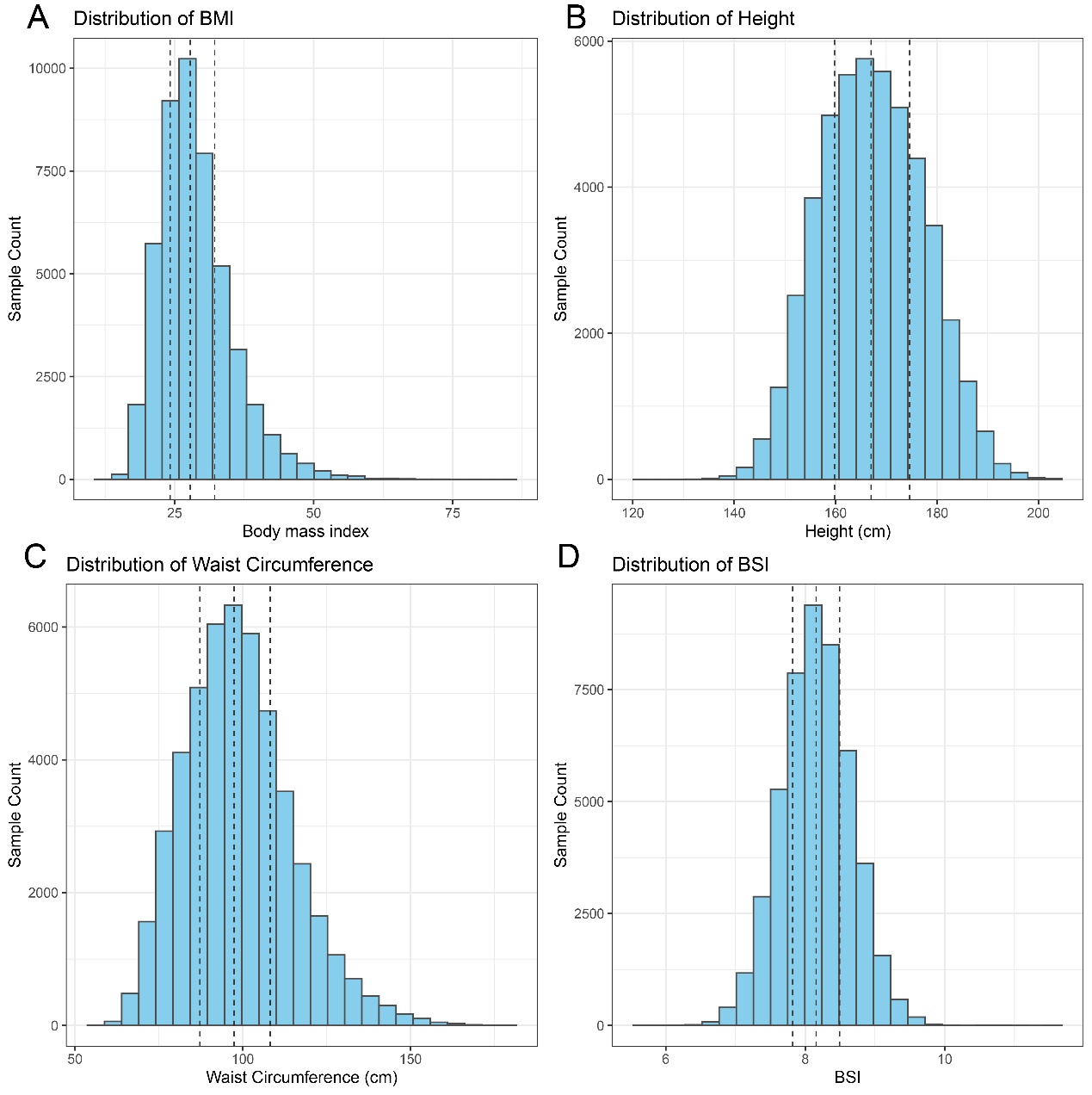


**Supplementary Figure 1**. Histograms of (A) BMI, (B) height, (C) waist circumference, and (D) BSI among included participants. Dashed lines indicate the median and interquartile range for each variable.


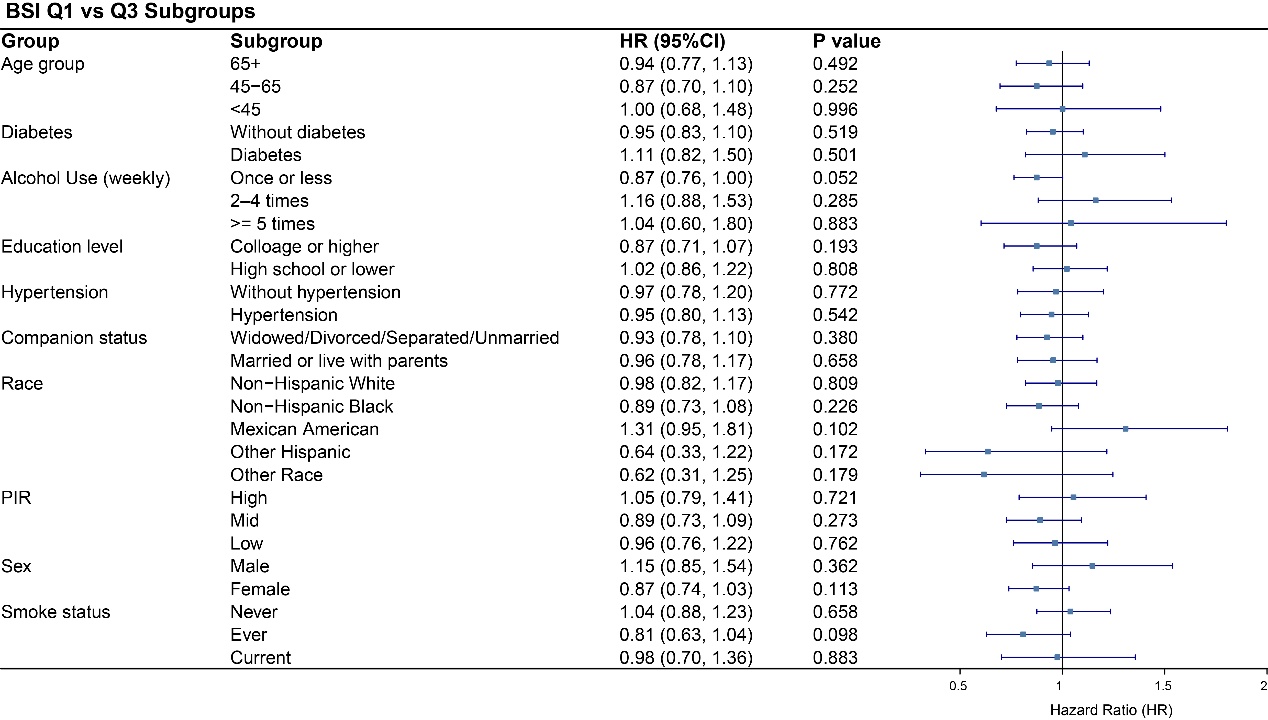


**Supplementary Figure 2**. Subgroup analyses of the association between the lowest BSI quintile (Q1) and the reference group (Q3) with all-cause mortality. Hazard ratios (HRs) and 95% confidence intervals are shown for each subgroup; all comparisons were non-significant.
